# Dendritic Cells From the Cervical Mucosa Capture and Transfer HIV-1 via Siglec-1

**DOI:** 10.3389/fimmu.2019.00825

**Published:** 2019-04-30

**Authors:** Daniel Perez-Zsolt, Jon Cantero-Pérez, Itziar Erkizia, Susana Benet, Maria Pino, Carla Serra-Peinado, Alba Hernández-Gallego, Josep Castellví, Gustavo Tapia, Vicent Arnau-Saz, Julio Garrido, Antoni Tarrats, Maria J. Buzón, Javier Martinez-Picado, Nuria Izquierdo-Useros, Meritxell Genescà

**Affiliations:** ^1^IrsiCaixa AIDS Research Institute, Badalona, Spain; ^2^Department of Biochemistry and Molecular Biology, Universitat Autònoma de Barcelona, Barcelona, Spain; ^3^Department of Infectious Diseases, Vall d'Hebron Institut de Recerca, Barcelona, Spain; ^4^Institut d'Investigació en Ciències de la Salut Germans Trias i Pujol, Badalona, Spain; ^5^Pathology Department, Hospital Universitari Germans Trias i Pujol, Badalona, Spain; ^6^Pathology Department, Hospital Universitari Vall d'Hebron, Barcelona, Spain; ^7^Department of Morphological Sciences, Universitat Autònoma de Barcelona, Barcelona, Spain; ^8^Hospital Municipal de Badalona, Badalona, Spain; ^9^Department of Obstetrics and Gynecology, Hospital Universitari Germans Trias i Pujol, Badalona, Spain; ^10^University of Vic-Central University of Catalonia (UVic-UCC), Vic, Spain; ^11^Institució Catalana de Recerca i Estudis Avançats, Barcelona, Spain

**Keywords:** cervix, Siglec-1, HIV-1, *trans*-infection, myeloid cells

## Abstract

Antigen presenting cells from the cervical mucosa are thought to amplify incoming HIV-1 and spread infection systemically without being productively infected. Yet, the molecular mechanism at the cervical mucosa underlying this viral transmission pathway remains unknown. Here we identified a subset of HLA-DR^+^ CD14^+^ CD11c^+^ cervical DCs at the lamina propria of the ectocervix and the endocervix that expressed the type-I interferon inducible lectin Siglec-1 (CD169), which promoted viral uptake. In the cervical biopsy of a viremic HIV-1^+^ patient, Siglec-1^+^ cells harbored HIV-1-containing compartments, demonstrating that *in vivo*, these cells trap viruses*. Ex vivo*, a type-I interferon antiviral environment enhanced viral capture and *trans*-infection via Siglec-1. Nonetheless, HIV-1 transfer via cervical DCs was effectively prevented with antibodies against Siglec-1. Our findings contribute to decipher how cervical DCs may boost HIV-1 replication and promote systemic viral spread from the cervical mucosa, and highlight the importance of including inhibitors against Siglec-1 in microbicidal strategies.

## Introduction

Women account for 51% of people living with HIV worldwide, and in 2017 this represented 18.8 million of females (1). Since HIV-1 infection is mostly acquired by sexual transmission (2), understanding the female genital tract immunobiology is imperative not only to halt novel infections, but also to design strategies that will limit HIV-1 spread within the mucosa and contain the virus during the early stages of infection. HIV-1 acquisition requires a series of orchestrated events that lead to systemic infection, beginning with viral entry through the genital epithelium and followed by the productive infection of distinct CD4^+^ target cells that reside within the mucosa. Local infection is early disseminated to draining lymph nodes, aiding to spread HIV-1 systemically and boost viral replication (3). While current antiviral agents are potent inhibitors of viral infection in the mucosa, efficacy of preventive methods is also critically dependent on effective blockade of all potential receptors involved in HIV-1 dissemination from the genital mucosa to the lymphoid tissues (4–6). Yet, the precise molecular mechanisms underlying viral dissemination routes from the genital mucosa that boost systemic HIV-1 infection remain unknown.

Although productive infection during the first days after vaginal SIV exposure is low and mainly restricted to the cervicovaginal tissues (3), evidence for rapid dissemination to the draining lymph nodes has been demonstrated as soon as 24 h post-infection (7–9). These studies suggest a critical role for cervical myeloid cells and, particularly, dendritic cells (DCs) in the early dissemination of mucosal viruses to lymphoid tissues. Importantly, viral spread does not only rely on *de novo* productive infection of myeloid cells (10–12), but can be triggered upon activation of mucosal myeloid cells via the capture and storage of large amounts of HIV-1 particles that are later transferred to target cells, as previously reported for monocyte-derived DCs (13–15). Once mucosal myeloid cells migrate to secondary lymphoid tissues for induction of antiviral immune responses, trapped viruses can be efficiently transferred to CD4^+^ T cells (10, 11), which become productively infected and fuel systemic viral dispersion. This highly infectious process is known as *trans*-infection, and pioneering work already identified that migratory HLA-DR^+^ CD3^−^ cervical cells efficiently captured and transmitted HIV-1 in *trans* (5). Recent data also demonstrated that among all myeloid cell subsets, vaginal and cervical DCs capture and transport transmitted/founder viruses through the cervicovaginal mucosa and facilitate infection of target cells (16, 17).

Although *trans*-infection was initially attributed to the capacity of C-type lectin receptors such as DC-SIGN to specifically bind to the viral envelope glycoprotein of HIV-1 (11), viral capture in the female genital mucosa is independent of this particular receptor, as the majority of cervical DCs capturing HIV-1 do not express DC-SIGN (18) and neutralizing antibodies against DC-SIGN cannot block viral transmission (5). In addition to C-type lectin receptors, HIV-1 capture by myeloid cells is mediated by the sialic acid binding immunoglobulin-like lectin-1 (Siglec-1/CD169) (19–21). This receptor potently enhances HIV-1 capture and storage in virus containing-compartments (22), that are later released from DCs to infect target cells via virological synapse formation (23). Siglec-1 is present on the surface of myeloid cells and its expression is potently enhanced by type-I interferon signaling (21, 24), which is triggered by viral immune sensing or bacterial lipopolysaccharide exposure, both factors associated to HIV-1 infection (25). However, it does not bind to the envelope glycoprotein of HIV-1 as C-type lectin receptors do (11), but recognizes sialylated gangliosides that are anchored on the viral membrane of enveloped retroviruses (19, 26). *In vivo*, Siglec-1 is required for robust infection and early dissemination of a retrovirus within the lymphoid tissue of a murine model (27), but its potential role during HIV-1 infection in the female genital tract remains unexplored.

Here we sought to clarify if *trans*-infection mediated by Siglec-1 could impact the early stages of HIV infection in women, and performed a comprehensive analysis of Siglec-1 expression on human cervical anatomical compartments. In all samples studied, we identified mucosal DCs expressing this receptor, whose expression was boosted by interferon alfa (IFNα) antiviral signaling. Moreover, we also found that cervical DCs enhanced viral capture and *trans*-infection, and that this mechanism was effectively prevented with antibodies against Siglec-1 receptor.

## Results

### Myeloid Cells From Human Cervical Submucosa Express Siglec-1

**Figure 1:**
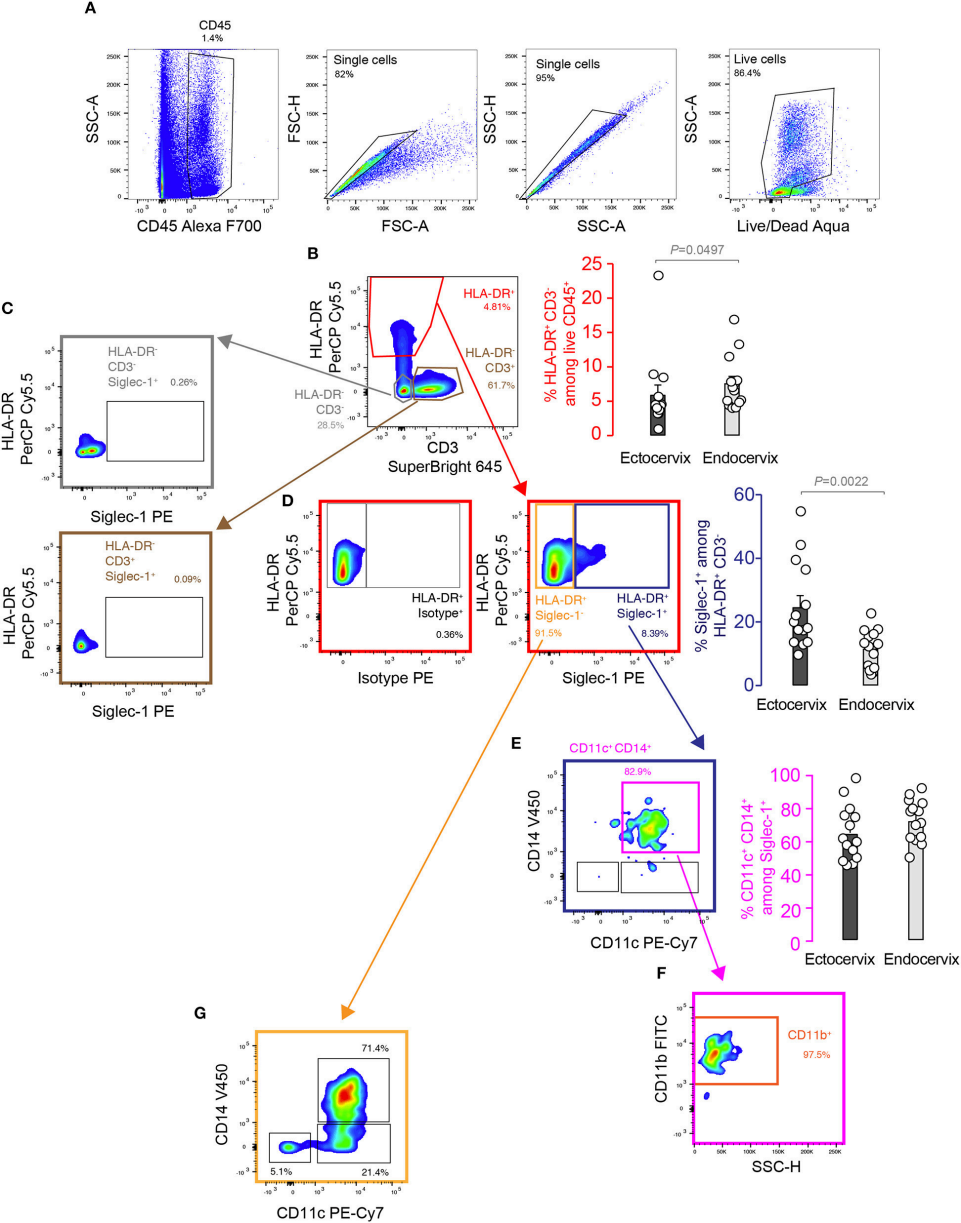
Myeloid cells from human cervical mucosa express Siglec-1. FACS analysis and representative gating strategy of the cervicovaginal myeloid cell subsets. Colored gates and arrows indicate populations analyzed, and corresponding matching colors are used to identify bar graphs showing frequencies of those populations. **(A)** Hematopoietic cells were identified by their CD45 expression, and single-live cells were selected by doublet discrimination and live/dead staining. **(B)** Representative dot plot and frequency of HLA-DR^+^ and CD3^−^ on hematopoietic cells. **(C)** Representative dot plot showing that HLA-DR^−^ cells do not express Siglec-1. **(D)** Representative dot plot and frequency of Siglec-1 expression analyzed in HLA-DR^+^ myeloid cells compared to matched isotype control. **(E)** Representative dot plot and frequency of CD11c^+^ CD14^+^ cells among myeloid HLA-DR^+^ cells expressing Siglec-1. **(F)** Representative dot plot of CD11b expression analyzed in CD11c^+^CD14^+^ Siglec-1^+^ myeloid cells. **(G)** Representative dot plot of CD14 and CD11c among the myeloid HLA-DR^+^ cells that do not express Siglec-1. All bar graphs show mean values and SEM from 14 donors. Statistical differences were assessed with a Mann-Whitney test.

We first analyzed Siglec-1 expression by flow cytometry on human cervical mononuclear cells obtained from tissues from benign hysterectomies processed immediately after prescribed surgery. Gating on hematopoietic CD45^+^, single and viable cells allowed identifying myeloid CD3^−^ HLA-DR^+^ cells (Figure 1A), which represented a mean of 6 and 7.5 % of cells at the ectocervix and endocervix, respectively (red gate and bar graph, Figure 1B). Of note, HLA-DR^−^ cells did not express Siglec-1 (gray and brown gates, Figure 1C). However, among the myeloid HLA-DR^+^ fraction, a mean of 24.5 and 11.5 % of cells expressed Siglec-1 in the ectocervix and endocervix, respectively (blue gate and bar graph, Figure 1D). Moreover, these cells were predominantly CD11c^+^ CD14^+^ (pink gate and bar graph, Figure 1E) and CD11b^+^ (orange gate, Figure 1F). In contrast, myeloid HLA-DR^+^ cells lacking Siglec-1 expression represented three distinct subsets: CD11c^−^ CD14^+^, CD11c^+^ CD14^−^ and CD11c^+^ CD14^+^ cells (Figure 1G). Of note, Siglec-1^+^ cells displayed a higher activation status than Siglec-1^−^ cells as seen by HLA-DR expression levels (Supplemental Figure 1). These results identify Siglec-1^+^ myeloid cells at the ectocervix and endocervix as a subset that displays typical markers of interstitial cervical DCs, such as HLA-DR, CD11c, CD14, and CD11b (28).

### Siglec-1^+^ Cells Accumulate in the Submucosa of the Ectocervix and Endocervix

**Figure 2:**
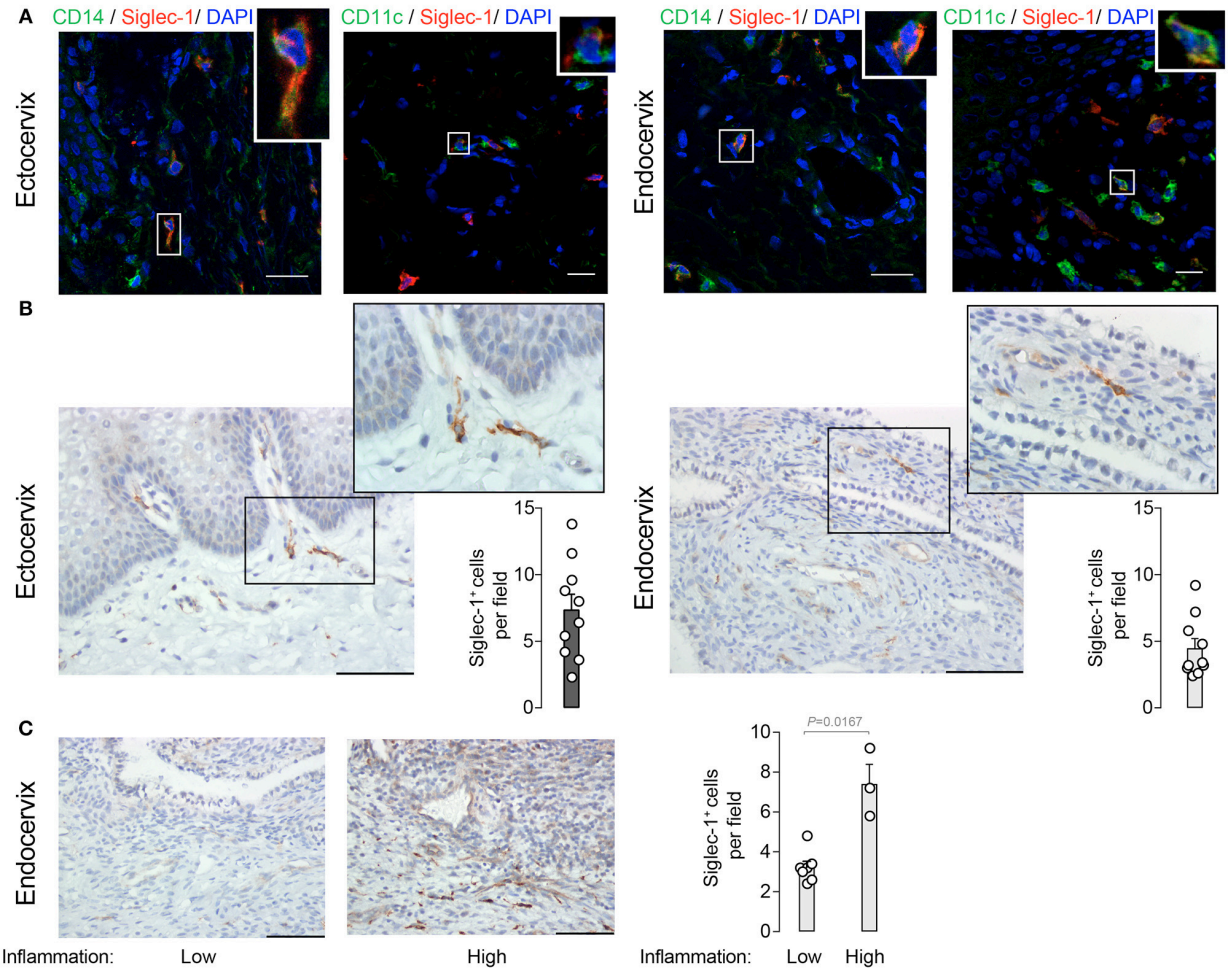
Siglec-1^+^ cells accumulate in the submucosa of the ectocervix and endocervix. **(A)** Representative immunofluorescent stainings on four ectocervix and endocervix of CD14 (AF488) and Siglec-1 (AF647), or CD11c (AF488) and Siglec-1 (AF647). Scale bars 20 μm. **(B)** Representative images of Siglec-1 immunostaining (40x) on 10 ectocervix and endocervix. Scale bars 100 μm. Insets show Siglec-1^+^ cells magnification (100x). Bar graph shows the mean values and SEM of Siglec-1^+^ cells per field counted in 5 consecutive fields. **(C)** Representative images of Siglec-1 immunostaining (40x) on 10 endocervix displaying a low and high grade of inflammation. Scale bars 100 μm. Bar graph shows the mean values and SEM of Siglec-1^+^ cells per field counted in 5 consecutive fields of 10 endocervix with different inflammation grades. Statistical differences were assessed with a Mann-Whitney test.

To analyze the localization of Siglec-1^+^ cells within the cervix, we next performed immunofluorescence of mucosal tissues from women undergoing benign hysterectomies. Siglec-1^+^ cells predominantly located in the lamina propria or submucosa and, in agreement with flow cytometry data, were also positive for CD14 or CD11c (Figure 2A). In contrast, no Siglec-1^+^ cells were found in the lower region of the epithelium, where CD207/langerin^+^ Langerhans cells are commonly found (29). Immunohistochemistry staining of cervicovaginal tissues further identified Siglec-1^+^ cells displaying myeloid cell morphology mostly accumulated within the submucosa of the ectocervix, which is lined by a stratified squamous epithelium (Figure 2B, left). Siglec-1^+^ cells were also found in the endocervix, which is covered only by a single-layer columnar epithelium (Figure 2B, right), although the frequency was lower than at the ectocervix. However, in those endocervical tissues classified as highly inflamed based on histopathological detection of inflammatory infiltrates, we found a higher number of Siglec-1^+^ cells (Figure 2C) that were comparable to those observed at the ectocervix (Figure 2B, left graph). Of note, the reasons for a higher inflammatory infiltrate could not be attributed to the HIV-1 infection status, the level of viral load, age or any particular clinical indication for surgery (Table 1). These results identified the presence of myeloid cells expressing Siglec-1 in the steady state just beneath the mucosa of the ectocervix and endocervix, where cervical DCs usually accumulate (16).

**Table 1:** Clinical data associated to cervical tissues where Siglec-1 was quantified by IHQ.

**Patient**	**Siglec-1^**+**^ cells per field at the Ectocervix**	**Siglec-1^**+**^ cells per field at the Endocervix**	**Age (yr)**	**Indication for surgery**	**HIV status**	**Viral Load (copies/ml)**
1	9,6	2,6	52	Prolapse	-	NA
2	2,3	3,4	53	Prolapse	-	NA
3	4,2	7,2^*^	45	Prolapse	-	NA
4	13,8	9,2^*^	52	Prolapse	-	NA
5	3,6	5,8^*^	60	Prolapse	-	NA
6	8,8	4,8	59	Unknown	+	< 50 (ART)
7	6,4	2,4	45	Prolapse	+	< 50 (ART)
8	11,6	3,2	57	Cystocele	+	140 (ART)
9	5,4	3	41	Uterine fibroids	+	400 Controller (naïve)
10	8	3,2	52	Prolapse + Cystocele	+	1.164 (naïve)

Higher levels of inflammation were observed in these tissues^*^

### DCs From Cervical Mucosa Mediate Viral Uptake via Siglec-1 and Are Detected *in vivo*

**Figure 3:**
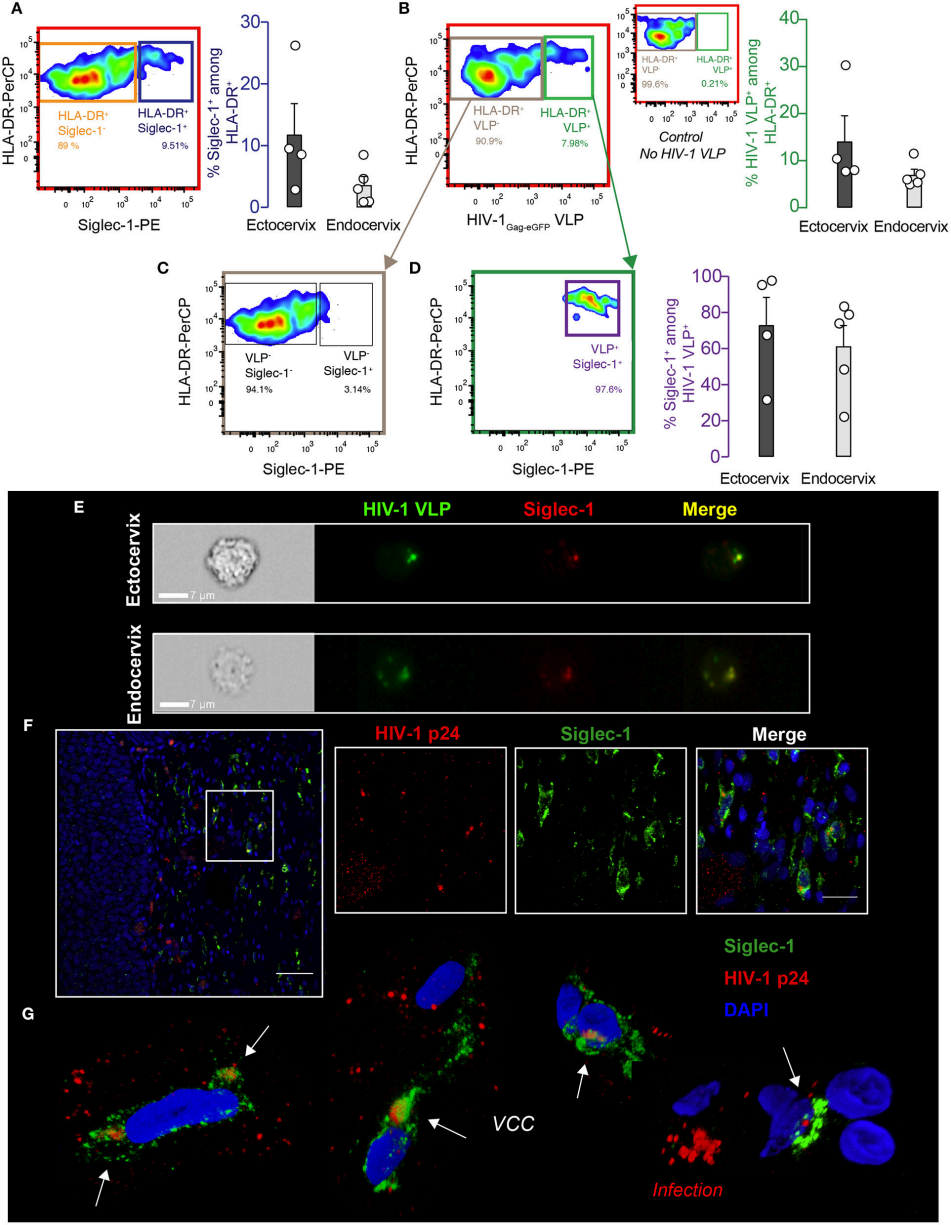
DCs from cervical mucosa mediate viral uptake via Siglec-1 and are detected *in vivo*. **(A)** Cervical mononuclear cells isolated from the ectocervix and endocervix of benign hysterectomies were pulsed with VLPs for 18 h at 37°C, extensively washed, labeled with the indicated mAbs and assessed by FACS. Colored gates and arrows indicate populations analyzed, and corresponding matching colors are used to identify bar graphs showing frequencies of those populations. Representative dot plot and frequency of HLA-DR^+^ and Siglec-1^+^ cells on hematopoietic cervical cells. **(B)** Representative dot plot and frequency of cells capturing HIV-1_Gag−eGFP_ VLPs among the myeloid HLA-DR^+^ fraction. Smaller dot plot in between depicts the control without VLPs. **(C)** Representative dot plot showing reduced expression of Siglec-1 in the myeloid HLA-DR^+^ cells not capturing HIV-1_Gag−eGFP_ VLPs. **(D)** Representative dot plot of Siglec-1^+^ cells among the cells capturing HIV-1_Gag−eGFP_ VLPs. Bar graphs show mean values and SEM from the ectocervix and endocervix of 4 to 5 donors. **(E)** Images of Siglec-1^+^ cervical cells pulsed and labeled as in **(A)**. Cells were acquired by Amnis-imaging FACS, and showed green fluorescent HIV-1_Gag−eGFP_ VLPs accumulation within a sac-like virus-containing compartment enriched in Siglec-1 (labeled in red). **(F)** Paraffin-embedded cervical tissue from one viremic HIV-infected woman stained for HIV-1 p24 antigen (labeled in red), Siglec-1 (in green), and nucleus (in blue). Scale bar 50 μm. (Inset panels) zoom in of squared region with distinct fluorescences (scale bar 20 μm). **(G)** 3D volumetric x-y-z data fields reconstruction of Siglec-1^+^ cells from four distinct areas of the cervical tissue of the viremic HIV-infected woman. Opacity representation of DAPI stained nuclei and fluorescence of the sac-like virus-containing compartment (VCC; white arrows). Right bottom image displays a characteristic cell pattern with p24^+^ dots reflecting viral production (Infection).

To investigate whether Siglec-1^+^ cervical myeloid cells could capture and store HIV-1 via the interaction with this particular I-type lectin receptor, we next pulsed cervical cell suspensions processed immediately after prescribed surgery with fluorescent viruses. Cells were incubated with HIV-1 Virus-Like Particles (HIV-1_Gag−eGFP_ VLPs), which lack the viral envelope glycoprotein but carry sialyllactose-containing gangliosides recognized by Siglec-1 receptor and recapitulate HIV-1 uptake results obtained with wild type viruses in myeloid cells (30, 31). After VLP exposure, cervical mononuclear cells were extensively washed and assessed by flow cytometry. Among the single-live CD45^+^ myeloid HLA-DR^+^ fraction, a mean of 11.8 and 3.6 % of cells expressed Siglec-1 in the ectocervix and endocervix (blue gate and bar graph, Figure 3A), while a 14.1 and 7 % of cells captured HIV-1_Gag−eGFP_ VLPs (green gate and bar graph, Figure 3B). Moreover, cells not capturing VLPs lacked Siglec-1 expression (gray gate, Figure 3C), while cells that captured viruses were predominantly Siglec-1^+^ in most of the tissues (purple gate and bar graph, Figure 3D). Viral uptake by Siglec-1^+^ cells was further confirmed by Amnis-imaging FACS technology, which allowed microscope inspection of acquired cells and screening of Siglec-1/VLP co-localization. Amnis viral capture assays revealed that fluorescent HIV-1_Gag−eGFP_ VLP localized with Siglec-1 within a virus-containing compartment (Figure 3E), resembling to the sac-like structures previously described in monocyte-derived DCs and activated tonsillar myeloid cells (19, 31). As HIV-1_Gag−eGFP_ VLP had no viral envelope glycoprotein, observed uptake could only rely on Siglec-1 recognition of sialylated gangliosides anchored on viral membranes (26, 30). Thus, Siglec-1^+^ myeloid cells found in the ectocervix and endocervix capture HIV-1_Gag−eGFP_ VLP in a virus-containing compartment via Siglec-1 recognition of viral membrane gangliosides.

To further assess if Siglec-1^+^ cervical cells are capable of capturing wild type HIV-1 during the natural course of infection, we next investigated the cervical biopsy of a viremic HIV-1^+^ patient by immunostaining. Confocal microscopy analysis revealed that distinct Siglec-1^+^ cells found in the cervical submucosa harbored viral p24 antigens (Figure 3F). Three-dimensional z-stack reconstructions of Siglec-1^+^ cells from distinct tissue areas showed virus-containing compartments that were different from the p24 staining of productively infected cells lacking Siglec-1 expression (Figure 3G and Movie 1). Thus, Siglec-1^+^ cervical cells can trap viruses throughout the course of HIV-1 infection *in vivo*.

### pDCs Exposed to HIV-1 Induce Siglec-1 Expression on DCs via IFNα Secretion

**Figure 4:**
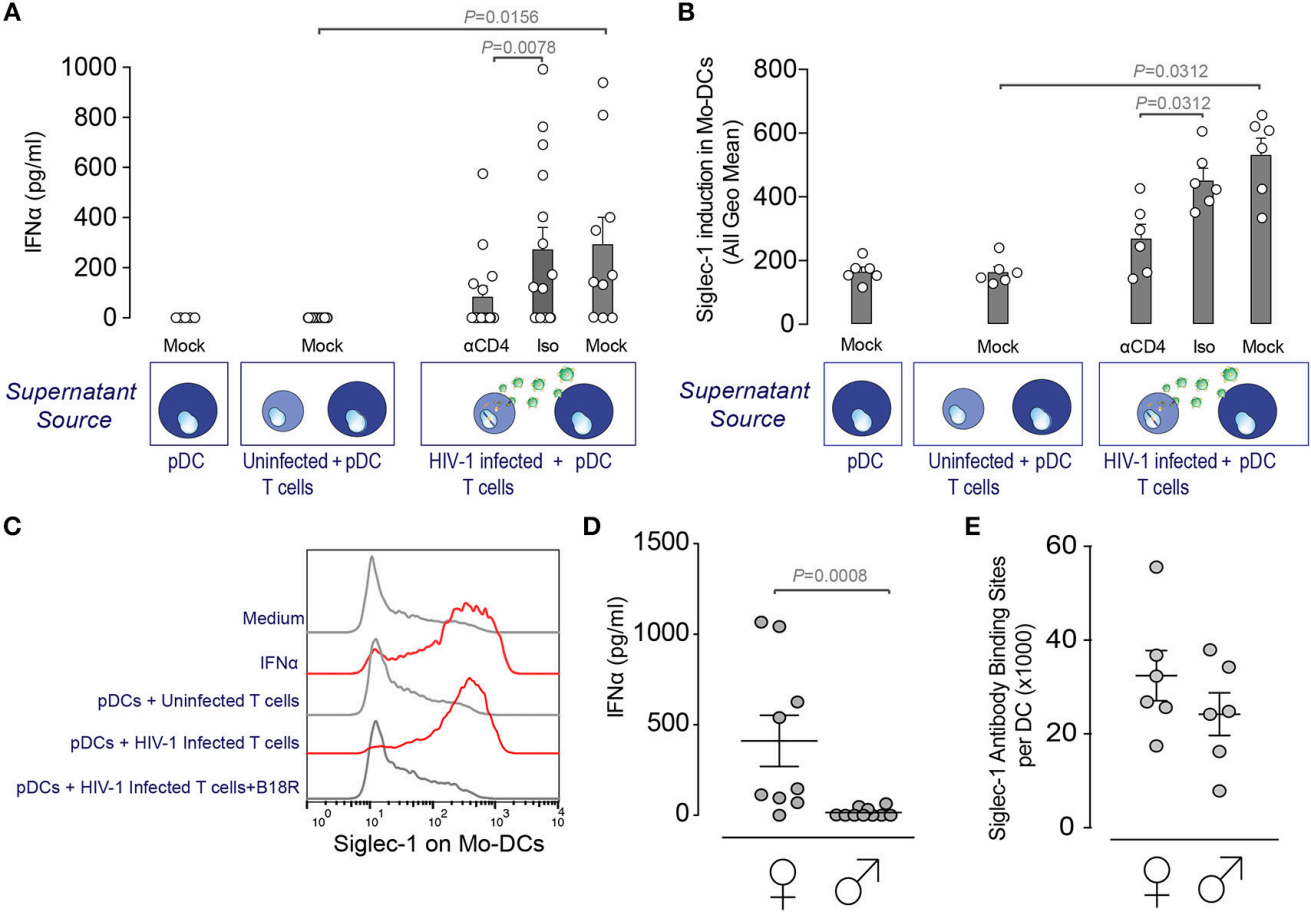
pDCs exposed to HIV-1 induce Siglec-1 expression on DCs via IFNα secretion. **(A)** IFNα release measured by ELISA on supernatants from blood derived pDCs co-cultured 24 h alone, with an uninfected MOLT CD4^+^ T cell line or an HIV-1 infected MOLT CD4^+^ T cell line that chronically produces R5-tropic BaL viruses in the presence or absence of 10 μg/ml of an anti-CD4 or an isotype mAb. Bar graph shows mean values and SEM from at least 6 donors and 3 independent experiments. Statistical differences were assessed with a Wilcoxon matched-pairs signed rank test. **(B)** Induction of Siglec-1 on monocyte-derived DCs incubated with supernatants isolated from pDCs co-cultured as in **(A)** and assessed by FACS. **(C)** Representative histograms of Siglec-1 expression on monocyte-derived DCs exposed to medium, recombinant IFNα, supernatants from uninfected pDCs, supernatants from HIV-1-exposed pDCs on mock treated DCs or on DCs previously incubated with the type-I interferon blocking receptor B18R. **(D)** IFNα release measured by ELISA on supernatants of pDCs isolated from women or men and co-cultured 24 h with an HIV-1 infected MOLT CD4^+^ T cell line. Bar graph shows mean values and SEM from 19 donors and 4 independent experiments. Statistical differences were assessed with a Mann-Whitney test. **(E)** Quantification of Siglec-1 expression levels on monocyte-derived DCs from men and women assessed by FACS. Bar graph shows mean values and SEM from 12 donors and 3 independent experiments. Prentice Rank Sum Test was used to assess statistical differences, which did not reach statistical significance (*P* = 0.073).

Although the basal expression of Siglec-1 on cervical DCs already allows viral uptake, the level of expression of this type-I interferon inducible receptor (21) correlates with viral capture and transmission efficiency of DCs (20). Hence, right after HIV-1 infection and once the antiviral type-I IFN responses are triggered, we hypothesized that IFNα could up-regulate Siglec-1 expression on cervical DCs and enhance their viral uptake capacity. In the cervical mucosa, resident myeloid cells quickly induce type-I IFN responses after HIV/SIV infection (32). Moreover, plasmacytoid dendritic cells (pDCs), the most potent producers of IFNα, are soon recruited to the site of initial retroviral infection (33). Thus, we next assessed if IFNα secreted by HIV-1-exposed pDCs could trigger Siglec-1 expression on DCs, mimicking early events of HIV-1 pathogenesis once infection has been established and type-I IFN antiviral immunity is mounted. Blood derived pDCs were cultured alone, co-cultured with an uninfected T cell line or with an HIV-1-infected T cell line that chronically produces R5-tropic BaL viruses in the presence or absence of anti-CD4 or isotype monoclonal antibodies (mAb). HIV-1-exposed pDCs released higher IFNα compared to non-exposed pDCs (Figure 4A), while blockage of pDC infection with a mAb against CD4 reduced IFNα secretion compared to an isotype control (Figure 4A). Accordingly, Siglec-1 expression was increased on monocyte-derived DCs incubated with supernatants from HIV-1-exposed pDCs as compared to supernatants from uninfected pDCs (Figure 4B), and further inhibited by an anti-CD4 mAb (Figure 4B). Thus, HIV-1 sensing via viral fusion on pDCs induced IFNα secretion that triggered Siglec-1 expression on monocyte-derived DCs as potently as recombinant IFNα (Figure 4C), and was specifically inhibited by the type-I interferon blocking receptor B18R (Figure 4C). Moreover, HIV-1-exposed pDCs derived from pre-menopause women released significantly higher amount of IFNα than pDCs derived from men (Figure 4D). We also induced Siglec-1 expression with equal amounts of recombinant IFNα on monocyte-derived DCs from men and women and determined the absolute number of Siglec-1 antibody binding sites per cell (Figure 4E). Under these experimental conditions, however, DCs derived from women did not significantly express higher amounts of Siglec-1 than DCs derived from men (Figure 4E). All together, these results highlight how upon HIV-1 infection, pDC accumulation in the female genital tract could lead to an increased antiviral IFNα secretion that could limit viral infection initially, but also promote viral capture of cervical myeloid cells via Siglec-1 induction.

### IFNα Enhances Viral Capture and *Trans*-infection on Siglec-1^+^ Cervical DCs

**Figure 5:**
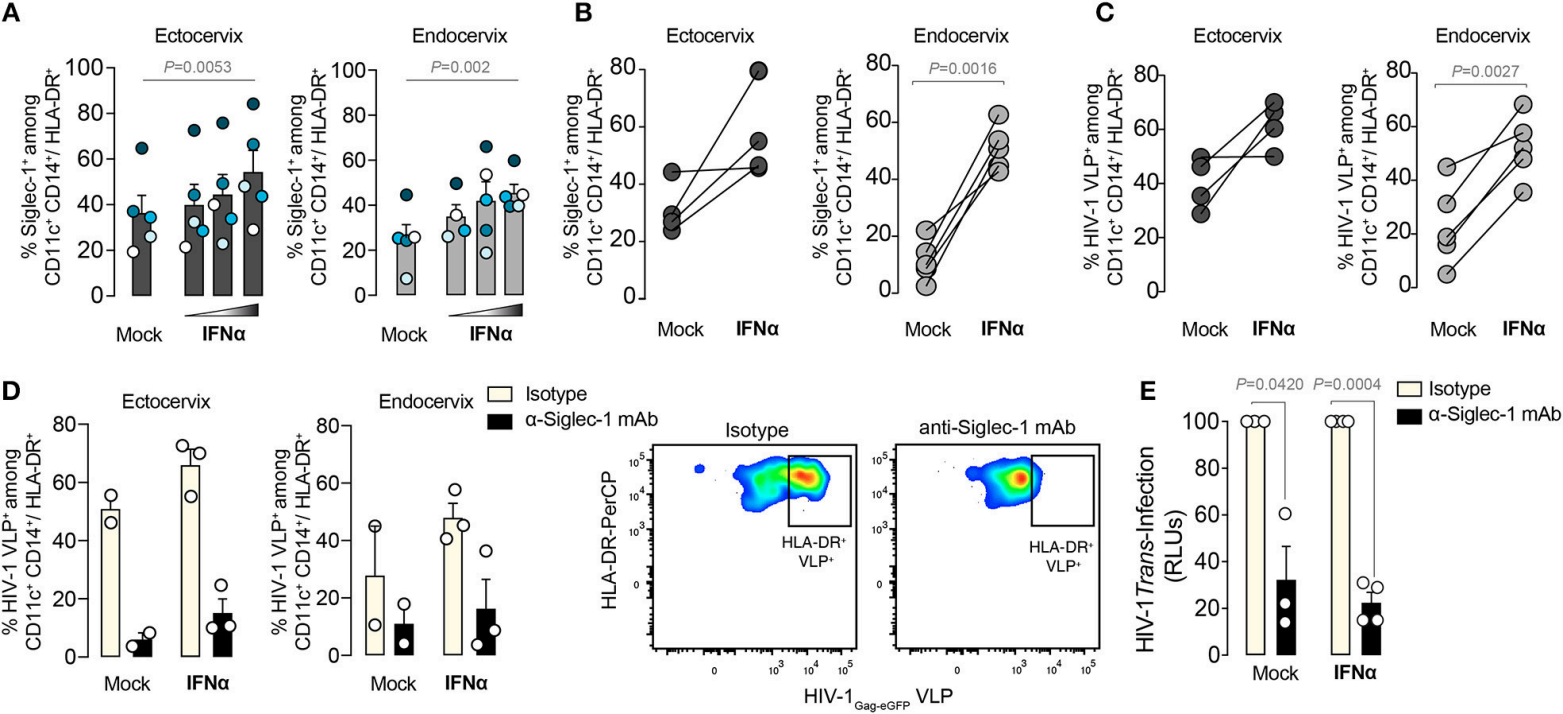
IFNα enhances viral capture and *trans*-infection on Siglec-1^+^ cervical DCs. **(A)** Percentage of Siglec-1^+^ DCs within the myeloid HLA-DR^+^ CD14^+^ CD11c^+^ fraction from small pieces of ectocervix or endocervix cultured in the presence or absence of increasing concentrations of IFNα (1,000; 10,000; and 100,000 IU/ml) and assessed by FACS as in Figure 3. Bar graph shows mean values and SEM from 5 donors and 4 independent experiments. Colors depict each particular donor. Statistical differences were assessed with a one-way repeated measures ANOVA test. **(B)** Percentage of Siglec-1^+^ DCs within the myeloid HLA-DR^+^ CD14^+^ CD11c^+^ fraction from cellular suspensions obtained from tissue digestion and cultured in the presence or absence of IFNα. Statistical differences were assessed with a paired *t*-test. **(C)** Percentage of cells capturing HIV-1_Gag−eGFP_ VLPs among the myeloid HLA-DR^+^ CD14^+^ CD11c^+^ fraction from cellular suspensions obtained from ectocervix or endocervix digestion and cultured in the presence or absence of IFNα. Statistical differences were assessed with a paired *t*-test. **(D)** Percentage of cells capturing HIV-1_Gag−eGFP_ VLPs as in **(C)** on cells that had been previously pre-incubated with 20 μg/ml of 7D2 anti-Siglec-1 mAb or isotype control. Bar graph shows mean values and SEM from 2-3 donors. Dot plots showing representative inhibition are also depicted. **(E)** Relative R5 tropic HIV-1_NFN−SX_ transmission to CD4^+^ target cells from cervical CD45^+^CD3^−^CD19^−^ HLA-DR^+^ sorted cells pre-incubated with 20 μg/ml of isotype or anti-Siglec-1 mAbs before viral exposure. Values are normalized to isotype-treated cells (set at 100%). Statistical differences were assessed with a one sample *t*-test. Mean values and SEM from two experiments include cells from 3 or 4 donors.

We next investigated if IFNα could trigger Siglec-1 expression on cervical myeloid cells and enhance their Siglec-1-mediated viral capture capacity. Small pieces of ectocervix or endocervix were cultured overnight (34), in the presence or absence of increasing concentrations of IFNα and analyzed by flow cytometry after enzymatic digestion. IFNα induced a dose-response increase in the percentage of Siglec-1^+^ cells among the myeloid HLA-DR^+^ CD14^+^ CD11c^+^ DC fraction (Figure 5A). Moreover, on cellular suspensions obtained from cervical tissues immediately processed after surgery, IFNα treatment also increased the percentage of Siglec-1^+^ cells (Figure 5B). This effect was more prominent in the endocervix, where basal expression of Siglec-1 was lower (Figures 1D, 5B). Accordingly, IFNα treatment increased the percentage of cells capturing HIV-1_Gag−eGFP_ VLP among the myeloid HLA-DR^+^ CD14^+^ CD11c^+^ DC population, especially at the endocervix (Figure 5C). Thus, endocervical myeloid cells could mediate *trans*-infection more potently once antiviral type I IFN responses are mounted and Siglec-1 expression is triggered on DCs.

Our results suggested that α-Siglec-1 mAbs could offer protection against HIV-1 uptake and prevent dissemination mediated by cervical DCs. We therefore pre-incubated cells from the ectocervix and endocervix with an α-Siglec-1 mAb or isotype control before viral exposure (Figure 5D). While isotype control had no inhibitory effect, pre-treatment with α-Siglec-1 mAbs led to a reduction of HIV-1_Gag−eGFP_ VLP uptake even after IFNα treatment (Figure 5D). Moreover, this blocking effect was further confirmed using a wild type R5-tropic HIV-1_NFN−SX_ to pulse mock treated or IFNα-treated CD3^−^HLA-DR^+^ cervical DCs, which were sorted and co-cultured with a reporter CD4^+^ cell line to measure viral transfer via *trans*-infection (Figure 5E). Of note, at the steady state, the expression of Siglec-1 on these cells is shown on Figure 1D. In seven independent cervicovaginal tissues, we consistently observed a significant decrease in the levels of HIV-1 transmitted in the α-Siglec-1 mAb treated cells compared to the isotype control, which accounted for blocking a mean of 70% of the transferred infectivity by myeloid cells. Thus, at the basal state but also upon Siglec-1 induction by IFNα, the α-Siglec-1 mAb could most likely block viral binding and uptake, preventing subsequent viral transmission from myeloid cells to target cells. These results highlight that specific inhibitors against Siglec-1 can halt viral transfer and dispersion mediated by Siglec-1^+^ cervical DCs.

## Discussion

A preventative strategy aimed at protecting against HIV infection must block several steps during the earliest stages of infection, avoiding not only the productive infection of primary target cells, but also the viral dissemination toward distal tissues. In both scenarios, antigen-presenting cells residing in different compartments of the female genital mucosa may play a prominent role (7, 9, 24, 35). DC-T cell conjugates represent an optimal milieu for productive HIV infection, which may boost initial viral replication of CD4^+^ T cells (10, 36). Moreover, uninfected DCs could promote HIV dissemination to draining lymph nodes after mucosal challenge (8, 9). Yet, the precise molecular mechanism mediating these processes remained unexplored in the cervicovaginal tissue.

Here we have shown the ability of Siglec-1 to mediate viral capture and transmission to target cells on a population of cervical myeloid cells expressing HLA-DR, CD11c, CD14, and CD11b, all classical markers known to be present on DCs of the submucosa at the lower female genital tract (18, 28, 29, 37). Our findings concur with prior studies where the migratory CD3^−^ HLA-DR^+^ fraction or the cervicovaginal CD11c^+^ DCs were found to be the myeloid cell subset that preferentially captured and transported HIV-1 out of the cervicovaginal mucosa (5, 16, 17). Nevertheless, we now show that on DCs of the ectocervix and endocervix, alternative receptors beyond DC-SIGN operate in HIV-1 viral uptake and transfer, as previously reported for monocyte-derived DCs (13, 15, 38–43). Our results provide a plausible mechanism for previous reports where the predominant cervical myeloid cell population that captured HIV-1 was found to be DC-SIGN negative (18), or where there was a lack of inhibition of neutralizing antibodies against DC-SIGN on viral transmission mediated by cervical myeloid cells (5). The discovery of Siglec-1 expression on myeloid cervical DCs and the capacity of Siglec-1^+^ cells to capture viruses *in vivo* help to understand how this particular receptor can facilitate boosting of HIV-1 replication and dissemination from the genital mucosa to the corresponding draining lymph nodes in the absence of DC productive infection.

Baseline levels of Siglec-1 on myeloid cells in the lamina propria of all cervical tissues examined herein already allowed viral uptake, demonstrating that Siglec-1 could act as a viral attachment factor even in the absence of prior viral infection. However, as tissues with a high level of inflammatory infiltrate showed an increased number in Siglec-1^+^ cells, ongoing inflammatory events triggered upon infection could magnify Siglec-1 mediated HIV-1 uptake and *trans*-infection. Indeed, in a cervical biopsy of an HIV-1 viremic woman, we found Siglec-1^+^ cells with HIV-1-containing compartments, demonstrating that these cells actually trap viruses and form these compartments *in vivo*.

In the female genital tract, type I IFN responses from tissue resident cells represent a potent first-line of defense against many pathogens, including lentiviruses (44–47). Soon after infection, recruited pDCs sensing incoming viruses will strongly contribute to IFNα secretion (33). Here we confirmed that HIV-1 fusion on pDCs induced IFNα secretion (48, 49) and that this mechanism, which is subjected to hormonal regulation (50), was enhanced in pDCs derived from pre-menopausic women. However, gender did not affect the IFNα response that triggered Siglec-1 induction on DCs, as it had been previously reported for other interferon-stimulated genes during chronic HIV-1 infection (51). Further, we demonstrated that CD14^+^ CD11c^+^ DCs up-regulate Siglec-1 expression in a dose response manner after IFNα stimulation of the cervical tissue, especially at the endocervix. In contrast, higher basal expression of Siglec-1 was found on ectocervical myeloid cells at the steady state. These data suggest that while pre-existing basal immune activation at the ectocervix could already favor Siglec-1 capture of early invading viruses, endocervical cells will most likely mediate viral uptake at later time points, once antiviral type I IFN responses are mounted and Siglec-1 expression is boosted on DCs. Indeed, this could explain why we only observed an increased number of Siglec-1^+^ cells on biopsies from the endocervix that had a high inflammatory score. Overall, detection of Siglec-1 on cervical DCs contributes to understand why the IFN system is not more effective against HIV-1 despite its substantial up-regulation early upon infection (4, 33, 52). Similarly, it may also explain why treatments inducing pDC recruitment (53) and a strong type I IFN response (54) before vaginal challenge in macaques are incapable of limiting viral infection beyond the infected mucosa and can even enhance viral replication.

As other sexually transmitted infections such as herpes virus or chlamydia infection trigger type I IFN responses in mucosal tissues via pDC recruitment and/or bacterial lipopolysaccharide exposure (55, 56), it would be important to explore the role of Siglec-1 in favoring HIV-1 acquisition, replication and dissemination in women with pre-existing sexually transmitted infections (2). Moreover, since inflammatory CD14^+^ CD11c^+^ DCs are known to induce Th17 T-cell differentiation (57), and these are the preferential targets of viral infection in the cervix right after retroviral invasion (58), it would also be critical to evaluate the role of this Siglec-1^+^ DC subset on susceptibility to HIV-1 infection.

In summary, myeloid DCs expressing a key molecule involved in HIV-1 *trans*-infection, namely Siglec-1, are found in the cervical tissues of women in the basal state. IFNα-treated myeloid DCs up-regulate Siglec-1 expression, which increases viral capture and *trans*-infection, providing an explanation of how the virus may succeed in an otherwise antiviral environment. We propose that Siglec-1^+^ cervical DCs may facilitate HIV-1 transfer to bystander CD4^+^ T cells and favor the nascent infection within the cervical mucosa, but also facilitate early dissemination to secondary lymphoid tissues. These findings highlight the importance of including Siglec-1 inhibitors along with potent antiretroviral agents in forthcoming microbicidal strategies, to stop not only the productive cellular infection in the cervix, but also the systemic viral dissemination from the female genital tract.

## Methods

### Cervical Tissue Digestion and Immunophenotype

Human cervical tissue was obtained from women (age range 39–82 years) undergoing hysterectomy for non-neoplasic indication at either HUGTiP or Hospital Municipal de Badalona. After confirmation of healthy tissue status by the Pathology Service, a piece from ectocervix and endocervix separated by anatomical localization was delivered to the laboratory in tissue media [RPMI 1640 supplemented with 15 % FBS, 500 U/mL penicillin, 500 μg/mL streptomycin, 2.5 μg/mL Fungizone (Life Technologies), 50 μg/mL gentamicin, MEM-non essential aminoacids (Gibco) and 1 mM MEM-sodium pyruvate (Gibco)]. Tissue was processed within the next 24 h after surgery. The mucosal epithelium and the underlying stroma of both ectocervix and endocervix were separated from muscular tissue and dissected into approximately 8-mm^3^ blocks. Depending on the experiment, tissue blocks were cultured, digested or included in optimal cutting temperature compound for immunofluorescence as previously described (34).

For tissue digestion, five to eight pieces from ectocervix or endocervix were separately placed into 1.5 ml-tubes containing 5 mg/ml collagenase IV (Invitrogen) in RPMI 1640 supplemented with 5% fetal bovine serum (FBS, Gibco). Tubes were shaked at 400 rpm at 37°C for 30 min. After enzymatic digestion, tissue blocks were dissociated manually with a disposable pellet pestle in two series of 20 rotations while pulling it up and down. The suspension was filtered through a 70 μm cell strainer (SPL Life Sciences), and cells were collected at 16°C after washing with PBS. Cells were then stained with Live/Dead Aqua (Invitrogen) for 30 min at RT. Pellet was suspended in staining buffer (1% mouse serum, 1% goat serum in PBS) and stained with CD14-V450 (MØP9), CD11c-PE-Cy7 (B-ly6), HLA-DR-PerCP-Cy5.5 (G46-6), (all from BD Biosciences), CD3-eVolve 655 (OKT3) (eBiosciences), CD45-Alexa Fluor 700 (Hl30), CD11b-FiTC (M1/70), Siglec-1-PE (7-239) (all from BioLegend). Mouse IgG1-PE (BioLegend) was used as isotype control. Cells were acquired using a BD LSRFortessa SORP flow cytometer (Flow Cytometry Platform, IGTP) and analyzed with FlowJo vX.0.7 software (TreeStar).

### Immunofluorescence

Cervical 5-μm cryosections were dried at RT, fixed in 3.7% formaldehyde (Sigma Aldrich) diluted in PHEM buffer as previously described in (34), permeated with 0.2% Triton X-100 (Sigma Aldrich) in PHEM buffer and blocked with 0.2% cold fish gelatin (Sigma Aldrich), 0.1% Triton X-100 and 10% normal goat serum (Gibco) diluted in PBS. Sections were first incubated with the following primary mAbs: mouse anti-Siglec-1 7-239 Ab (Abcam), rabbit anti-CD11c EP1347Y Ab (Abcam) or rabbit anti-CD14 EPR3653 Ab (Abcam). Samples were then washed extensively with PBS and incubated with the secondary mAbs Alexa 488-conjugated donkey anti-rabbit or Alexa 647-conjugated donkey anti-mouse IgG (Jackson ImmunoResearch). Sections were covered with mounting medium (ProLong™ Gold Antifade Mountant with DAPI, Life Technologies, Invitrogen) and a coverslip. Images were obtained by confocal microscopy using a Zeiss LSM 710 microscope and the Zen Blue Image acquisition software.

### Immunohistochemistry

Ten cervical formalin-fixed, paraffin-embedded samples from the Pathology Department of HUGTiP were analyzed. Of note, we chose samples from five HIV-1 infected and five seronegative women, but results were equivalent regardless of the HIV status. Immunohistochemical stains were performed using a Ventana Benchmark Ultra (Ventana Medical Systems) in accordance with the manufacturer's protocol, with standard antigen retrieval (pH 9.0; Ventana) and the mAb against α-Siglec-1 (clone SP213, LS Biosciences, dilution 1/100) for 12 min. A pathologist carried out a blind quantification of Siglec-1^+^ cells and images were captured using a DP71 digital camera (Olympus, Center Valley, PA, USA) attached to a BX41 microscope (Olympus). Siglec-1^+^ cells in five consecutive fields in the subepithelial area were counted for ectocervix and endocervix separately. Tissues where a significant inflammatory infiltrate was detected were considered as highly inflamed.

### Cell Lines

HEK-293T cells obtained from ATCC (CRL-11268) and TZM-bl (obtained through the US National Institutes of Health [NIH] AIDS Research and Reference Reagent Program) were maintained in Dulbecco's Modified Eagle Medium (DMEM). The human T cell lines MOLT CD4^+^ uninfected and MOLT CD4^+^ HIV-1_BaL_ infected have been described elsewhere (59) and were grown in RPMI. All media contained 10% FBS, 100 U/ml of penicillin and 100 μg/ml of streptomycin (all from Invitrogen).

### HIV-1_Gag-eGFP_ VLP and HIV-1 Generation

Fluorescent HIV-1 virus-like particles (HIV-1_Gag−eGFP_ VLP) were generated transfecting HEK-293T cells with plasmid pGag-eGFP obtained from the NIH AIDS Research and Reference Reagent Program. Replication-competent HIV-1 stock was generated by transfecting the proviral construct NFN-SX, an HIV-1_NL43_ provirus that expresses the HIV-1_JRFL_ envelope glycoprotein (kindly provided by W. O'Brien). Thirty micrograms of plasmid DNA were added to cells in T75 flasks, and transfection was performed using a calcium phosphate kit (CalPhos, Clontech). Forty-eight hours post-transfection, supernatants were harvested, filtered (Millex-HV, 0.45 μm; Millipore) and frozen at −80°C until use. The p24^Gag^ content of the VLP and HIV-1 stock was determined by ELISA (PerkinElmer). TCID_50_ or 50% tissue culture infective doses of HIV_NFN−SX_-infection used for *trans*-infection assays were determined by end-point dilution culture on the cell line TZM-bl, which contains an HIV long terminal repeat linked to a luciferase reporter gene.

### VLP Uptake Assays

1 × 10^6^ digested ectocervical and endocervical cells were pre-incubated for 15 min at RT with 20 μg/ml of an anti-Siglec-1 mAb (7D2) (Abcam), a murine IgG1 κ isotype control (BD Pharmigen) or left untreated. Cells were pulsed overnight with 2.7- 20 ng p24^Gag^ HIV-1_Gag−eGFP_ VLP at 37°C in a 5% CO_2_ incubator in the presence or absence of 1,000 IU/ml of recombinant Interferon-2α (Sigma-Aldrich) in 10% FBS RPMI with antibiotics. After extensive washing, cells were stained as previously described and acquired using a BD LSRFortessa SORP flow cytometer (Flow Cytometry Platform, IGTP) and analyzed with FlowJo v10.3 software (TreeStar).

For imaging flow cytometer analyses, 1 × 10^6^ digested ectocervical and endocervical cells were pulsed with HIV-1_Gag−eGFP_ VLP as previously described. After extensive washing, cells were resuspended in PBS with 1:250 Live/Dead Aqua (Invitrogen) and incubated for 30 min at RT. Cells were then fixed and permeabilized (Fix & Perm; Invitrogen), and stained with an anti-Siglec-1-PE mAb (7-239) (BioLegend). Cells were acquired with an Amnis ImageStreamX imaging flow cytometer (Merck), and analyzed using IDEAS v6.1 software. A gradient root mean square or RMS value > 40 was established as the best focus threshold, and single cells were selected in the Area vs. Aspect Ratio dot plot of the bright field channel.

### HIV-1 p24 Immunostaining

A cervical formalin-fixed, paraffin-embedded sample from a viremic patient diagnosed with sexually transmitted HIV-1 infection a decade ago was obtained from the Pathology Department of the University Hospital Vall d'Hebron. The sample was collected when the patient had between 5,160 and 10,400 HIV-1 RNA copies/ml in blood. Samples were de-waxed and placed in decreasing ethanol concentrations. Heat-induced epitope retrieval was performed in EDTA buffer pH 9 (Abcam) in a water bath at 100°C during 10 min. Slides were permeabilized with Tris-bufferd saline 1X (TBS) (Fisher scientific) with 0.1% Triton X-100 and 1% BSA (Sigma-Aldrich) for 10 min. Subsequently, blocking was performed with TBS 1x supplemented with 10% donkey serum (Jackson Immunoresearch) and 1% BSA for 2h. Mouse anti-p24 mAb (Kal-1; Dako-Agilent) was incubated overnight at 4°C. Later, rabbit anti-Siglec-1 mAb (SP213; Lifespan Bioscience) was incubated for 15 min at RT. Samples were then stained with secondary antibodies Donkey AlexaFluor 647 anti-mouse (Invitrogen) and Donkey AlexaFluor 488 anti-rabbit; counter stained with DAPI (Thermo Fisher) and mounted with Fluoromount G (eBioscience). Samples were imaged on an Olympus Spectral Confocal Microscope FV1000 using a 20x and 60x phase objective and sequential mode to separately capture the fluorescence at an image resolution of 800 × 800 pixels. Image J software was used for image processing. Alternatively, samples were acquired in z-stacks every 0.2 μm on a Zeiss LSM 780 confocal inverted microscope with an apochromatic 63x oil (NA = 1.4) and processed with Volocity software using the 3D Opacity module for reconstruction.

### Primary Cell Cultures

Peripheral blood mononuclear cells (PBMCs) were obtained from buffy coats of the *Banc de Sang i Texits* of Barcelona by Ficoll-Hypaque density gradient centrifugation (Alere Technologies AS). Plasmacytoid DCs were negatively isolated using magnetic beads from the Plasmacytoid Dendritic Cell isolation kit (Miltenyi Biotech) and immediately used for experiments. Monocytes were isolated using CD14^+^ selection magnetic beads (Miltenyi Biotech) and differentiated into monocyte-derived DCs with 1,000 IU/ml of granulocyte-macrophage colony-stimulating factor plus 1,000 IU/ml of Interleukin-4 (both from R&D) during 5 days before supernatant exposure. Cells were maintained in RPMI supplemented with 10% FBS, 100 U/ml of penicillin and 100 μg/ml of streptomycin.

### IFNα Release on Supernatants From pDCs and Siglec-1 Induction

A total of 0.1 × 10^6^ pDCs were co-cultured with 0.1 × 10^6^ HIV-1_BaL_-infected MOLT-4 cells for 24 h at 37°C. Before co-culture, some pDCs were also pre-treated with 10 μg/ml of anti-CD4 mAb (clone RPA T-4) to avoid viral fusion or with an isotype mAb control (both from Beckton Dickinson) for 10 min at RT. As a negative control, pDCs were co-cultured with an uninfected MOLT-4 cell line. After 24 h of co-culture, supernatants were collected and assessed for IFNα production with VeriKine Human IFN Alpha Elisa Kit (pbl Assay Science). Alternatively, supernatants from these co-cultures were transferred to 0.2 × 10^6^ DCs to assess Siglec-1 induction 24 h later with a FACSCalibur, labeling cells with a mAb anti-Siglec-1-PE or a matched isotype-PE control (both from AbD Serotec). Of note, these supernatants were also added to DCs that had been previously incubated with 2 μg/ml of carrier-free recombinant B18R protein (eBioscience) to block type I IFN receptor. DCs were also cultured in the presence of RPMI media or 1,000 IU/ml of recombinant Interferon-2α. The mean number of Siglec-1 Ab binding sites per monocyte-derived DC from men and women was obtained with a Quantibrite kit (Becton-Dickinson) as previously described (13).

### Cervical Tissue Stimulation With IFNα

After dissection of the tissue as previously described, five pieces from ectocervix or endocervix were separately placed into a 12-well plate containing 1 ml of tissue culture medium. Interferon-2α was added to the medium at 1,000; 10,000 or 100,000 IU/ml. After 24 h at 37°C in 5% CO_2_, tissue was digested and the remaining culture plate was treated with accutase (Thermo Fisher Scientific) for 30 min at 37°C to detach adherent cells. Finally, tissue and adherent cells were pooled together and stained for flow cytometry as previously described.

### *Trans*-infection Experiments

Ectocervical and endocervical blocks from HIV-1 non-infected donors were left untreated or incubated overnight with 10,000 U/ml of IFNα and 100 ng/ml of CCL19 (Mip-3β). Tissue was then digested, pooled together and stained with mAbs as previously described to sort single CD45^+^CD3^−^CD19^−^HLA-DR^+^ live cells by FACS. Recovered cells were pre-incubated with anti-Siglec-1 7D2 or isotype control mAbs for 10 min at RT. Cells were subsequently incubated with 185 ng of p24/ml of an R5 tropic HIV-1_NFN−SX_ (with an estimated TCID_50_ of 116.824) in the presence of 20 μg/ml of the indicated mAbs for 4 h at 37°C. After extensive washes, myeloid cells were co-cultured with the reporter TZM-bl cell line at a 1:1 ratio for 48 h. Luciferase activity was measured with Britelite plus (Perkin Elmer) in a Synergy MX luminometer (Biotek).

### Statistical Analysis

Data are reported as the mean and the standard error of the mean (SEM) for each condition. We analyzed mean changes using Mann-Whitney test, Wilcoxon matched-pairs signed rank test, paired *t*-test, and one-way repeated measures ANOVA test as indicated, which were considered significant at *P* < 0.05. Significant mean changes from 100% of the data normalized to percentages were assessed with a one sample *t*-test, considered significant at *P* < 0.05. Gender main effect inference across multiple experiments was assessed using the Prentice Rank Sum Test, a generalized Friedman rank sum test with replicated blocked data. All analyses and figures were generated with the GraphPad Prism v7 Software and R v3.5.

## Ethics Statement

Informed written consent was obtained from all participants and the study protocols were approved by the University Hospital Germans Trias i Pujol (HUGTiP, Badalona, Spain) and the Vall d'Hebron University Hospital (HUVH, Barcelona, Spain) Clinical Research Ethics Committee (reference numbers PI-14-070 and PR (IR)294/2017). The study was undertaken in accordance with the Declaration of Helsinki and the requirements of Good Clinical Practice.

## Author Contributions

DP-Z, JC-P, NI-U, and MG conceived and designed the experiments. AH-G, JC, GT, JG, and AT obtained, processed, and analyzed tissues. DP-Z, JC-P, IE, SB, MP, CS-P, and VA-S performed the experiments. DP-Z, JC-P, IE, SB, MP, CS-P, JC, GT, MJB, JM-P, NI-U, and MG analyzed and interpreted the data. DP-Z, JC-P, NI-U, and MG wrote the paper.

### Conflict of Interest Statement

A patent application based on this work has been filed (US 62/832,054). The authors declare that no other competing financial interests exist and that the research was conducted in the absence of any commercial or financial relationships that could be construed as a potential conflict of interest.

## Supplementary Material

Movie 1 3D volumetric x-y-z data field reconstruction of a Siglec-1^+^ cell from the cervical tissue of a viremic HIV-infected woman. Opacity representation of DAPI stained nuclei and fluorescence of the sac-like virus-containing compartment along with Siglec-1 staining.

Supplemental Figure 1 Representative histograms of HLA-DR expression comparing the Siglec-1 positive and negative populations on CD3^−^ CD11c^+^ CD14^+^ cells from the ectocervix and endocervix. Bar graphs show the geometric mean fluorescence values and SEM of HLA-DR expression from 14 donors. Statistical differences were assessed with a Wilcoxon matched-pairs signed rank test.
