# Supplementary figures and images for: Dendritic Cells From the Cervical Mucosa Capture and Transfer HIV-1 via Siglec-1
Source: Front Immunol. 2019 Apr 30;10:825. doi: 10.3389/fimmu.2019.00825 (PMC6503733; doi:10.3389/fimmu.2019.00825)

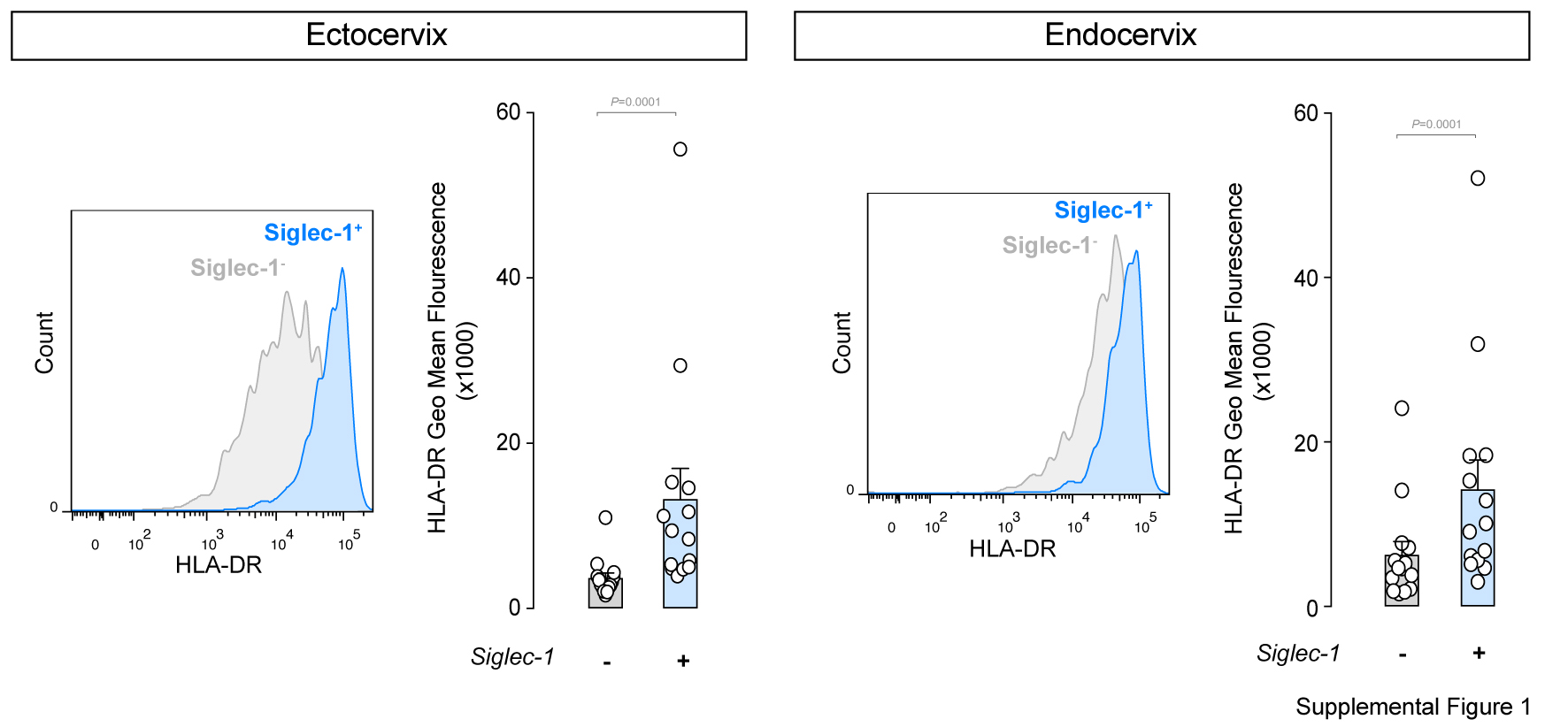

Supplement: Supplemental Figure 1 — Representative histograms of HLA-DR expression comparing the Siglec-1 positive and negative populations on CD3− CD11c+ CD14+ cells from the ectocervix and endocervix. Bar graphs show the geometric mean fluorescence values and SEM of HLA-DR expression from 14 donors. Statistical differences were assessed with a Wilcoxon matched-pairs signed rank test. [file Image_1.JPEG]
